# Chikungunya Virus Strains, Reunion Island Outbreak

**DOI:** 10.3201/eid1210.060596

**Published:** 2006-10

**Authors:** Maël Bessaud, Christophe N. Peyrefitte, Boris A.M. Pastorino, Fabienne Tock, Olivier Merle, Jean-Jacques Colpart, Jean-Sébastien Dehecq, Romain Girod, Marie-Christine Jaffar-Bandjee, Pamela J. Glass, Michael Parker, Hugues J. Tolou, Marc Grandadam

**Affiliations:** * Institut de médecine tropicale du Service de santé des armées, Marseille, France;; † Walter Reed Army Institute of Research, Silver Spring, Maryland, USA;; ‡ Agence de la biomédecine, Lyon, France;; § Direction régionale des affaires sanitaires et sociales, Saint-Denis, Île de la Réunion, France;; ¶ Centre hospitalier départemental Félix-Guyon, Saint-Denis, Île de la Réunion, France;; # United States Army Medical Research Institute for Infectious Diseases, Fort Detrick, Maryland, USA

**Keywords:** Chikungunya Virus, Alphavirus, La Reunion, Indian Ocean, Arbovirus, letter

**To the Editor:** Chikungunya virus (CHIKV) is endemic in rural tropical Africa and is penetrating urban areas in Asia. CHIKV is maintained in a sylvatic cycle that involves mosquitoes of the genus *Aedes*, primates, and rodents. CHIKV infection induces fever, arthralgia, and maculopapular rash. Hemorrhagic complications have been reported in some outbreaks, but a more specific symptom is severe arthralgia, often persistent, which results in long-lasting disability.

After numerous cases of CHIKV infection had been reported in Comoros and Mauritius (*1*), an outbreak of febrile illness was reported on Reunion Island in March 2005 (*2*). The incidence of the disease remained relatively low until December 2005, when it increased dramatically. The outbreak resulted in >3,500 confirmed cases and an estimated 250,000 suspected cases (*2*), affecting >25% of the island's inhabitants. Encephalitic forms were reported on many occasions during the active phase of the outbreak, and >200 persons died while they were infected with CHIKV. Previously unreported complications, such as mother-to-child transmission, myocarditis, hepatitis, and extensive dermal lesions were also encountered.

Many samples, collected from patients during the outbreak, were sent to our laboratory (Virology Unit, Tropical Institute of the French Armed Forces Medical Service, Marseille, France) to identify the etiologic agent. Serum samples incubated with C6/36 cells according to previously published methods (*3*) yielded CHIKV. This virus was also isolated from cerebrospinal fluid collected from a patient with encephalitis, from corneas collected from asymptomatic human organ donors, and from pools of mosquitoes (*Aedes albopictus* and *Culex quinquefasciatus*) collected on the island.

Five isolates were partially sequenced. The CHIKV genome was partly amplified by using the specific primer pair OP16/OP17 (*4*), and reverse transcription (RT)-PCR products (1,200 nucleotides long) were cloned and sequenced (GenBank accession nos. DQ462746–DQ462750). Comparison of partial sequences showed a high degree of identity between the strains isolated in Reunion, including the strain LR2006_OPY1 (*5*): paired identity was 99.3%–100% at the nucleotide level and 98.2%–100% at the amino acid level. The nucleotide and amino acid substitutions were homogeneously distributed across the sequence and were different for each isolate. Our strain IMT/6470, isolated from human serum, and the strain LR2006_OPY1 displayed the same nucleotide sequence in the sequenced region. The sequence identity among these isolates highlights the common origin of human and mosquito isolates.

The sequences of our isolates did not feature any codon deletions or insertions when compared with other CHIKV isolates from Africa and Asia available in GenBank (*4**,**6*). Strains from Reunion were also compared with the candidate vaccine strain TSI-GSD-218 (*7*). This strain showed 93%–94% and 96%–97% identity at the nucleotide and amino acid level, respectively, which suggests a sufficient antigenic community. Nevertheless, cross-neutralization experiments are necessary to confirm the protective effect of this candidate vaccine against Reunion strains.

In the phylogenetic tree based on the partial E1 sequences (Figure), all CHIKV strains isolated in Reunion clustered together. These strains were closely related to strains from the Central African Republic and the Democratic Republic of Congo (*4**,**6*). This finding suggests that the boundaries of the Central African CHIKV strains now extend to the Indian Ocean. The phylogenetic tree also illustrates the difference of lineage between the Reunion Island isolates and the Asian isolates.

**Figure:**
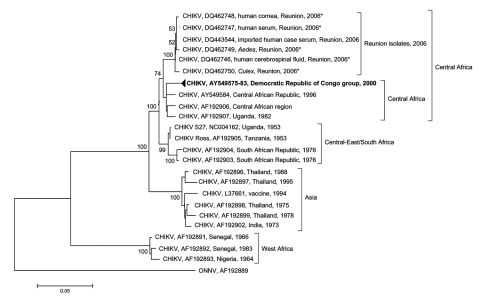
Phylogenetic tree of chikungunya virus (CHIKV) based on partial nucleotide sequences (3´ extremity of E1/3´ untranslated, position 10238–11367). Phylogram was constructed with MEGA 2 (http://megasoftware.net/mega2.html), and the tree was drawn with the Jukes-Cantor algorithm for genetic distance determination and the neighbor-joining method. The percentage of successful bootstrap replicates (1,000 bootstrap replications, confidence probability >90%) is indicated at nodes. The length of branches is proportional to the number of nucleotide changes (percentage of divergence). Asterisks (*) indicate strains isolated in this study. The dark triangle corresponds to viruses of the Democratic Republic of Congo clustering together (GenBank accession nos. AY549575–AY549583). O'nyong-nyong virus (ONNV) sequence has been introduced for correct rooting of the tree.

CHIKV has been isolated from *Culex* spp. collected during outbreaks (*8*), but laboratory experiments have shown that *Cx. quinquefasciatus* failed to transmit CHIKV to monkeys (*9*). Inside the Reunion cluster, the strain from *Culex* spp. was localized in a separate branch (bootstrap value 100%); this finding could be relevant to the different role of these mosquito species in virus epidemiology.

To our knowledge, CHIKV has never been isolated from human corneas. In our study, the cornea sample was obtained from an asymptomatic donor whose serum contained immunoglobulin M (IgM) but not IgG to CHIKV; this finding suggests the patient was recently infected with CHIKV. The presence of CHIKV in corneal cells will have to be confirmed because the samples we studied also included sclera, vascular tissue that could contain circulating virions; however, no virus was detected in the patient's blood sample by CHIKV-specific RT-PCR assay (*10*). Infected corneal or scleral cells may constitute a sanctuary that allows virus to persist after virus is no longer present in blood. Because viral persistence, which could explain long-lasting clinical complications of CHIKV infection, has never been demonstrated, this question deserves more investigation.

Our results indicate that CHIKV strains responsible for the outbreak in Reunion have a common origin and do not differ from strains circulating in East and Central Africa. More complete characterization of the 5 strains we report here, sequencing of the full-length genome, and phenotypic characterization of other CHIKV isolated in the area during the same period is currently underway in our laboratory.
